# Numerical research on the anisotropic transport of thermal neutron in heterogeneous porous media with micron X-ray computed tomography

**DOI:** 10.1038/srep27488

**Published:** 2016-06-08

**Authors:** Yong Wang, Wenzheng Yue, Mo Zhang

**Affiliations:** 1 State Key Laboratory of Petroleum Resources and Prospecting, China University of Petroleum, Beijing 102249, People’s Republic of China

## Abstract

The anisotropic transport of thermal neutron in heterogeneous porous media is of great research interests in many fields. In this paper, it is the first time that a new model based on micron X-ray computed tomography (CT) has been proposed to simultaneously consider both the separation of matrix and pore and the distribution of mineral components. We apply the Monte Carlo method to simulate thermal neutrons transporting through the model along different directions, and meanwhile detect those unreacted thermal neutrons by an array detector on the other side of the model. Therefore, the anisotropy of pore structure can be imaged by the amount of received thermal neutrons, due to the difference of rock matrix and pore-filling fluids in the macroscopic reaction cross section (MRCS). The new model has been verified by the consistent between the simulated data and the pore distribution from X-ray CT. The results show that the evaluation of porosity can be affected by the anisotropy of media. Based on the research, a new formula is developed to describe the correlation between the resolution of array detectors and the quality of imaging. The formula can be further used to analyze the critical resolution and the suitable number of thermal neutrons emitted in each simulation. Unconventionally, we find that a higher resolution cannot always lead to a better image.

Porous media, such as carbonate rocks, may have strong heterogeneity and anisotropy in pore structure. Neutron radiography is an effective non-destructive method, which is extremely sensitive to the element of hydrogen^1^,^2^. Therefore, it is often used to detect properties of porous media^3^,^4^,^5^ related to the hydrogen element, such as the distribution of water and mineral contents in ceramic, brick and concrete. Particularly, thermal neutrons play a critical role in identifying oil and water in the rock and soil^3^,^6^. However, very few literatures have been published to evaluate the anisotropy of pore structure with thermal neutrons. Besides, it is generally believed that there is no effect of the anisotropy of pore structure on the total porosity calculated with neutron data. Therefore, very few researches have been done to investigate the anisotropic transport of thermal neutrons due to the difficulty of studying the anisotropy in the traditional rock experiment. Compared with rock physics experiments, digital rock physics (DRP)^7^ has lots of advantages. With a high resolution of complex pore geometry, DRP has rapidly emerged as a quick and effective method to analyze properties of the rock core. Many numerical methods, such as finite element and finite difference methods, are used to calculate static elastic and electrical properties of digital core^8^,^9^,^10^. The Lattice-Boltzmann method is established to compute pore-scale fluid flow simulation^11^,^12^,^13^. Both acoustic properties and nuclear magnetic resonance (NMR) properties of digital core^14^,^15^, can be investigated by numerical simulation too. However, little attention has been focused on studying radioactive properties, since only the binary segmentation of matrix and pore^7^,^16^ has been taken into account in conventional models, which fail to set up the distribution of chemical multi-components. For thermal neutron transport, we have to consider the reaction between thermal neutrons and material contents filling in matrix and pore, respectively. Thus, according to the real core sample, we develop a new method to reconstruct the digital core model representing not only the three-dimensional pore structure but also the material components. With this model, the Monte Carlo method can be used to simulate the transport process of neutrons for revealing the relationship between the radioactive properties and intrinsic pore structure of rock. Based on our results, we can further analyze whether anisotropy of the rock has effects on the evaluation of porosity calculated by measured data of the neutron transport testing. Most likely, it is the first time that the anisotropy of pore structure has been studied by the thermal neutrons based on X-ray CT in this paper.

In this paper, we use two categories of digital core samples obtained by micron X-ray CT^7^,^17^. One group of rock image volumes is constructed from the carbonate (pure limestone) sample with of the density of 2.63 *g* · *cm*^−3^. The other group is constructed from the pure sandstone with the density of 2.42 *g·cm*^−3^. The physical resolution of all digital core samples is 2.2046 *µm* per pixel, and the utilized facility is UltraXRM-L200. After cropping, we can get fourteen 400 × 400 × 400 carbonate cubes and a 400 × 400 × 400 sandstone cube for further property simulations. Chemical elements of real rock samples are very complex. So we only consider main elements in our model. For carbonate (pure limestone), we set the chemical components of matrix and pore-filling fluid to be CaCO_3_ and H_2_O, respectively. For sandstone, we set the chemical components of matrix and pore-filling fluid to be SiO_2_ and H_2_O, respectively. Components of matrix and pore fluid are evenly distributed in their own space respectively.

MRCS of polyatomic molecule for thermal neutrons can be written as:


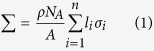


where Σ: macroscopic reaction cross section, *cm*^−1^;

*ρ*: density of molecule, *g* · *cm*^−3^;

*N*_*A*_: Avogadro constant, 6.02 × 10^23^;

*σ*_*i*_: reaction cross section of the *i*-th atom for thermal neutron in the molecule, barn (10^−24^ *cm*^2^);

*A*: molecular weight;

*n*: category number of atoms in the molecule;

*l*_*i*_ : number of the *i*-th atom in the molecule.

Table 1 shows the reaction cross section of 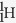, 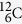, 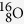, 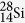 and 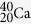 for thermal neutrons, the last column of Table 1 shows the element abundance in their isotopes. Generally, the primary elements in the first column have the maximum abundance which is commonly higher than 92%. Therefore, we only consider these primary elements in the model. Under one standard atmosphere pressure, densities of CaCO_3_, SiO_2_ and H_2_O are 2.71 *g·cm*^−3^, 2.65 *g·cm*^−3^ and 1.00 *g·cm*^−3^, respectively. The followings are MRCS of CaCO_3_, SiO_2_ and H_2_O for thermal neutron: 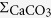 = 0.27 *cm*^−1^, 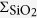 = 0.20 *cm*^−1^, 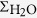 = 1.52 *cm*^−1^. MRCS of pore-filling fluid (H_2_O) for thermal neutron is much larger than that of matrix (CaCO_3_ and SiO_2_), because H_2_O contains more hydrogen. Therefore, as long as the material composition of matrix and pore-filling fluid is unchanged, the porosity has great influence on the transmission of incident neutrons^18^. Generally, the greater the porosity is, the smaller the transmittance is. Thus, the detected intensity distribution of thermal neutron changes with the porosity.

Figure 1 shows one example of the three-dimensional carbonate core sample, and an example of the three-dimensional sandstone core sample, respectively. Comparing Fig. 1(b,d), we can intuitively see that distributions of pore space in sandstone core are almost homogeneous in YZ, XZ and XY plane. However, all pore distributions in carbonate core are heterogeneous in the three orthogonal planes. There are obvious cracks distributed in XZ and XY plane of carbonate core (Fig. 1(b)), but not in YZ plane, which further demonstrates that pore distribution is strongly anisotropic.

According to the level of energy^19^, neutrons can be classified into cold neutron, thermal neutron, and epithermal neutron. Generally, the energy of thermal neutrons is 0.0253 *eV*. We construct the model for simulating those thermal neutrons irradiating core samples from a vertical direction of one surface plane. In order to record those unreacted thermal neutrons, the energy range of received neutrons by the array detectors is set between 0.0250 *eV* and 0.0256 *eV*. The thermal neutron source will randomly emit parallel thermal neutron beams with the energy of 0.0253 *eV* (the green part in Fig. 2). In each simulation, 40 million thermal neutrons are emitted into the model. When the thermal neutrons irradiate the core, some of them reacted with the core (mostly elastic scattering). Therefore, their energy and moving directions will change during the reaction. The thermal neutrons unreacted with the core, they can directly pass through the sample and reach to the array detectors. Thus, array detectors can record the distribution of unreacted thermal neutrons. Moreover, the neutron transmission imaging method would be used to obtain the distribution of difference of counts in YZ, XZ and XY plane, respectively.

## Results

### Thermal neutron imaging of the core

The selected carbonate core was irradiated with thermal neutron beam along directions of X, Y and Z, respectively. Figure 3(a,c,e) show the core’s porosity distribution in YZ, XZ and XY plane, respectively. Figure 3(b,d,f) show the corresponding difference of counts. As shown in Fig. 3, we respectively calculated the similarity of Fig. 3(a) VS Fig. 3(b,c) VS Fig. 3(d,e) VS Fig. 3(f). These similarities quantitatively verified that our method is quite effective. Figure 3(b) shows that the great difference of counts mainly appears in the middle and right part of the YZ plane, which is consistent with Fig. 3(a). Figure 3(d) shows that the pore’s distribution is uniform in XZ plane of the core. Figure 3(f) shows that there is a crack in XY plane of the core. Thus, according to Fig. 3(b,d,f), we can intuitively see that the distribution of pore varies obviously in different plane of the same core. This also demonstrates that the carbonate reservoir has strong heterogeneity and anisotropy.

### Analysis of the effect of core’s anisotropy

Ten cores were selected from the fourteen 400 × 400 × 400 carbonate cubes. After conducting the same simulation and data processing for each core along different directions, we can get corresponding difference of counts (such as Fig. 3(b,d,f)). Then the total difference of counts and the total porosity of each core can be calculated. The following equations are the linear fitting of relationship between total difference of counts and total porosity:


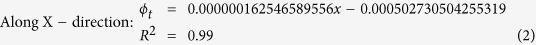



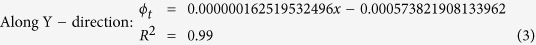



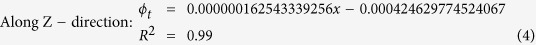


where *x* represents the total difference of counts obtained by the simulations of thermal neutron transporting along a given direction of each core, and *ϕ*_*t*_ represents the real total porosity of each core obtained from X-ray CT. Equation (2), (3) and (4) show the relationships between *ϕ*_*t*_ and *x* for the thermal neutron transporting along X-direction, Y-direction and Z-direction, respectively. *R*^*2*^ is the squared correlation coefficient. We can see that all the three fitting equations are consistent with the simulated data. In order to verify the three equations, we use the equations to predict the porosity of the rest four carbonate cores. The total difference of counts obtained by thermal neutron transporting along X-direction of each core is substituted into equations (2), (3) and (4) for calculating the porosity. Table 2 shows the results of our prediction.

In Table 2, the first column is the total difference of counts obtained by thermal neutron transporting along X-direction, and the second column is the real total porosity of each core obtained from X-ray CT. The next three columns represent the relative errors of prediction using equations (2), (3) and (4), respectively. By analyzing the last three columns, we can find that when we respectively substitute the same simulated data into the equations for porosity, the relative errors of the predicted results have small difference varying with the transport directions of neutron. Therefore, we can obtain a reasonable conclusion that the linear relationship between difference of counts and the porosity is affected by the anisotropy of core.

### The resolution of array detectors and the number of thermal neutrons emitted in every simulation

**Figure 1:**
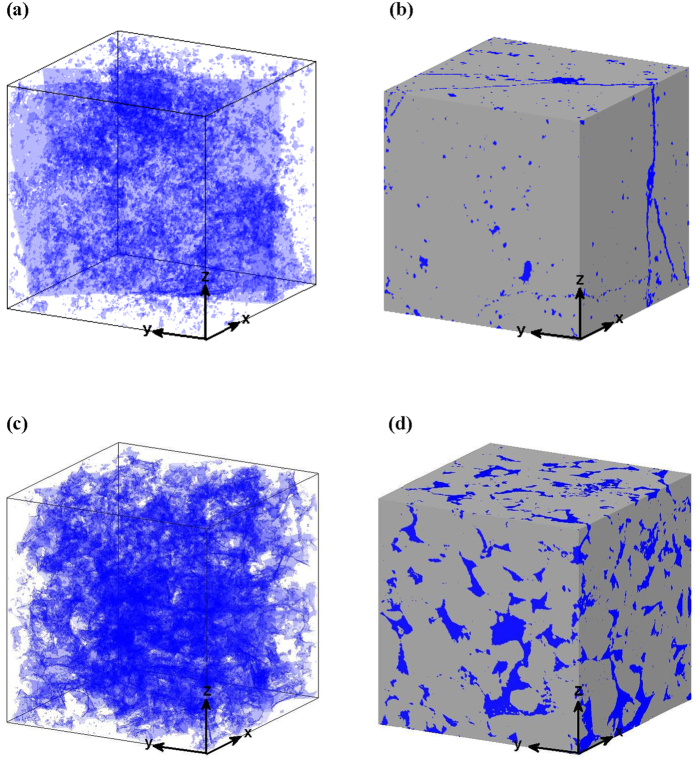
3D reconstructed pore structure of digital core based on micron X-ray CT. The total size of the dataset is 400 × 400 × 400 and the voxel edge length is 2.2046 *µm*. (**a**) Internal pore distribution of carbonate core, where the blue part is the pore structure; (**b**) Surface of the carbonate core; (**c**) Internal pore distribution of sandstone core, where the blue part is the pore structure; (**d**) Surface of the sandstone core.

**Figure 3:**
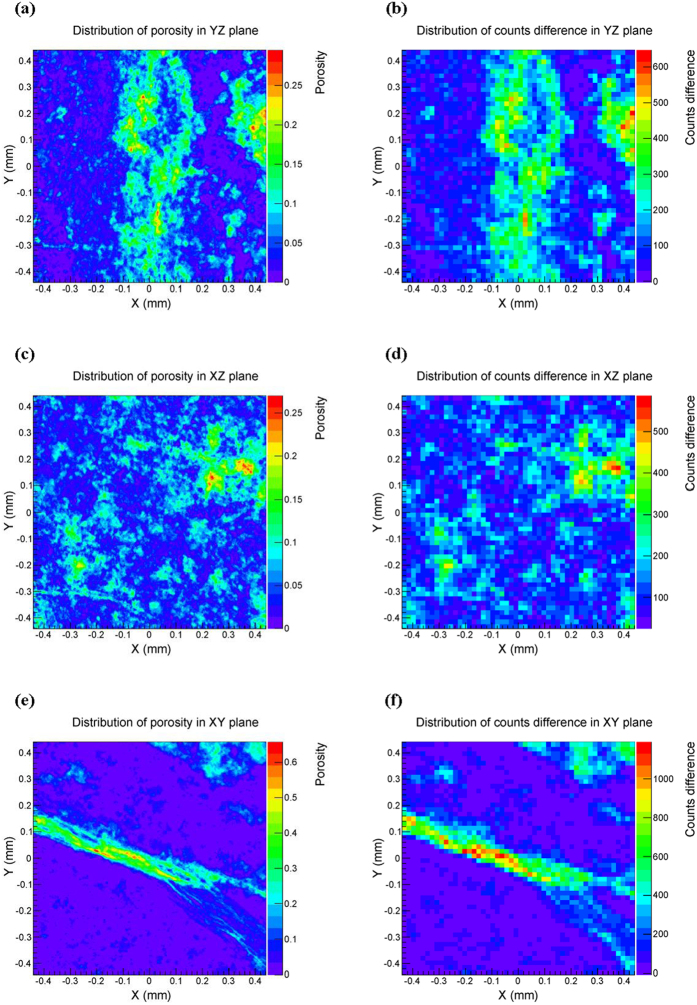
Thermal neutron imaging of the core. The similarity of (**a,b**) is 0.8385; the similarity of (**c,d**) is 0.7322; the similarity of (**e,f**) is 0.8063. The resolution of (**b,d,f**) is 17.6368 *µm*.

**Figure 4:**
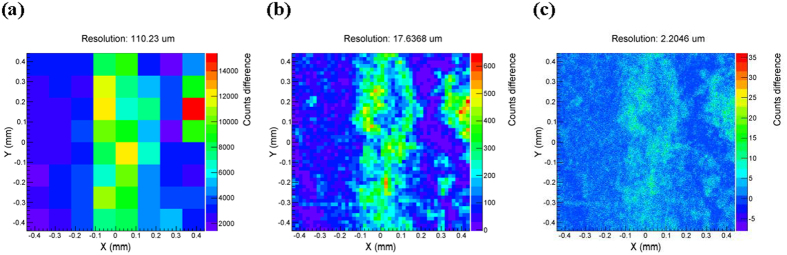
Detection results of carbonate core with different resolution.

**Figure 5:**
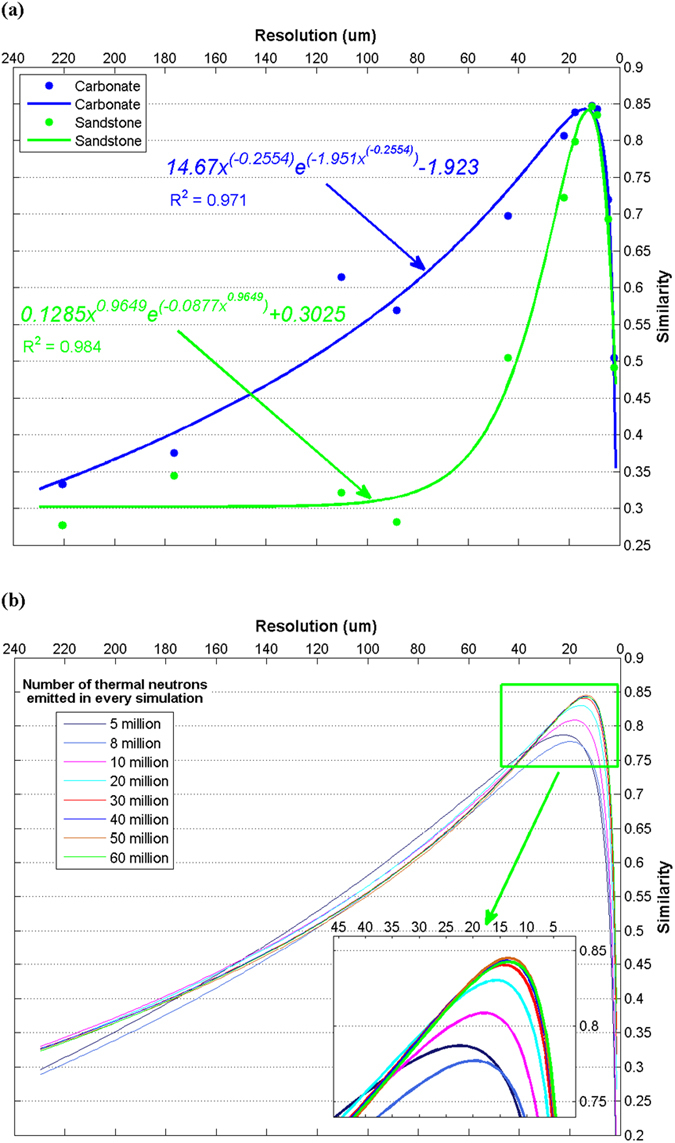
The resolution and the number of thermal neutrons emitted in each simulation.

Figure 3 shows that the transmission imaging method can correctly reveal the distribution of pores, cracks and fractures inside the rock. Accordingly, we can effectively analyze heterogeneity and anisotropy of the core with these images. During the simulation, the scale of array detectors varies from 4 × 4 to 400 × 400. The resolution depends on the edge length of each detector in the array. Thus, the dynamic range of resolution used in our research varies from 220.46 *µm* to 2.2046 *µm*, which allows us to investigate the effects of the resolution conveniently. On this basis, we further simulate transport of thermal neutron beam irradiating the carbonate core as shown in Fig. 1 along X-direction. After processing the data, we can get a series of detected images with different resolutions. Some of them are shown in Fig. 4. Comparing Figs 3(a) and 4, we can find that a higher resolution doesn’t always lead to a better image. In another word, there is a critical resolution. Moreover, we respectively calculated the similarity (correlation coefficient) between the original model and each detected image with different resolution. The calculated results had been plotted in Fig. 5(a), where the blue points and the blue line are for the carbonate, and the green points and the green line are for the sandstone. The abscissa in Fig. 5(a) represents the resolution of array detector, and the ordinate represents the similarity. From Fig. 5(a) we can see that the similarity will increase with the increasing resolution of array detectors, then reach a maximum and decrease rapidly after that. A new equation has been developed to describe the relationship between the similarity and the resolution, shown as,


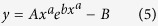


where *x* represents the resolution of array detector, and *y* represents the similarity, and *A*, *B*, *a*, *b* are constant parameters with related to the porous medium.

Specifically, the relation between the similarity and the resolution in Fig. 5(a) can be written as


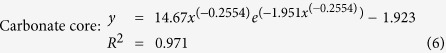



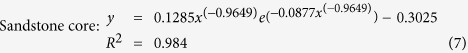


Obviously, the equation (5) is suitable for both carbonate and sandstone although they have different material compositions and pore distributions.

Such simulation for carbonate core has been repeated 8 times by changing the number of thermal neutrons emitted in each simulation. The relationships between the similarity and the resolution of all simulations are shown in Fig. 5(b). According to Fig. 5(b), we can observe that the trend of the relationship between resolution and similarity is similar with that in Fig. 5(a), although the number of thermal neutrons emitted in each simulation is different. There is a partially enlarged view of the green zone, as plotted in the lower right side of Fig. 5(b). It illustrates the variety of peak points of similarity with the number of thermal neutrons emitted in the simulation. Basically, the peak will rise when the number increases. However, when the number is more than 40 million, the peak point will stop increasing. Generally, the peak point only distributes in a range of the resolution between 10 *µm* and 20 *µm*. At present, the resolution of array detector for thermal neutron can reach 15 *µm*^20^. Therefore, according to our model with 40 million thermal neutrons emitted in the simulation, the resolution of array detectors used in our research is set to 17.6368 *µm* for carbonate core.

## Discussion

We developed a new method to reconstruct the digital core based on micron X-ray CT, which can consider not only the three-dimensional pore structure but also the material components. We simulated the transport of thermal neutrons in porous media, and analyzed the results for investigating the effects of anisotropy on the evaluation of porosity. According to the results, we can find that the method of thermal neutron transport can intuitively reveal the internal distribution of pores, cracks and fractures. We have drawn the conclusion that the linear relationship between the count difference and the total porosity is slightly affected by the anisotropy of the core. Therefore, when we use neutrons to detect the porosity of porous media, the porosity almost keeps the same value if we only change the direction of detection. In our models, the compounds or mixture distributed in matrix and pore are supposed to be uniform. And the pore-filling fluids, such as water, oil and natural gas, are the main material components containing hydrogen. Therefore, we can quantitatively detect the distribution of these substances. The conclusions may underlie anisotropic analysis of pore structure using the nuclear detection method. Currently, neutron radiography is often used to detect distribution of substances that contain hydrogen^4^. And our method could be of significance as a reference due to its flexible ability in handling complex pore structure and components.

We also draw the conclusion that when we investigate the pore distribution with thermal neutron transmission, a higher resolution of array detector cannot always lead to a better imaging. There is a critical resolution with a suitable number of thermal neutrons emitted in each simulation.

## Methods

### Reconstruction of the core based on micron meter X-ray CT

**Figure 6:**
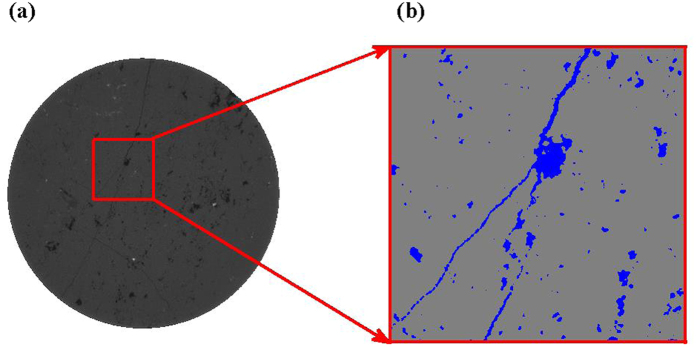
(**a**) A 2D cross-sectional view of carbonate tomographic image with 2.2046 *µm* voxel spacing; (**b**) Binary image, blue areas represent the pore, grey areas represent the matrix.

X-ray was firstly found by Wilhelm Röntgen in 1895, and X-ray CT was invented by Hounsfield and Cormack in 1979. Historically, X-ray CT was firstly applied in medical fields. Then it was extended to materials research and other fields^21^. X-ray CT^17^,^22^ is a non-destructive technique. It can reveal the internal structure of the core. When monochromatic X-ray transport through the medium, some parts of the X-ray will interact with the medium, leading to the attenuation of energy. Different components of the medium have different attenuation coefficients, as shown blow:


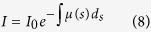


where *I*_0_ is the initial intensity of X-ray; *I* is the intensity of X-ray that transported through the medium; *μ(s)* is the local attenuation coefficient along the path of X-ray. By measuring the attenuation coefficient of substance for X-ray, we can determine the components of the substance. When a cylindrical rock sample has the diameter of a few millimeters or less, the resolution can be a few micron, or even better. In our model, the reconstruction process of the core includes three steps: image acquisition, image processing and 3D reconstruction. By using micron X-ray CT, we can get lots of raw radiographs (such as Fig. 6(a)). Then we need to process these raw radiographs (including noise reduction, smoothing, cropping and segmentation)^7^. Thus we can get segmented binary data (400 × 400) with matrix and pore voxels (Fig. 6(b)). With the binary data, we can reconstruct the three-dimensional core model which can consider not only the three-dimensional pore structure but also the material components. Moreover, the pore-filling material in the model is only H_2_O, and material of matrix is only CaCO_3_ or SiO_2_ for carbonate or sandstone respectively. Therefore, those components are not uniformly mixed according to the ratio of elements like the traditional methods. Instead, they are completely separated and unevenly distributed.

In order to reduce computer memory and the consumption time, the scale of the model should be optimized. The representative elementary volume (REV)^23^ method has been applied to implement the optimization by analyzing the variety of porosity of the model with the scale. After reaching the scale of REV, the porosity would keep stable if the model size continues increasing. REV scale can help to determine the minimum elementary volume to represent the whole rock. In this research, the scale of REV is chosen to be 400 × 400 × 400.

### Monte Carlo simulation

Monte Carlo method^24^,^25^ is used in this research to simulate the transport and scatter of thermal neutrons in the core model. Monte Carlo method is known as a random or statistical experimental method. This simulation method can realistically describe the physical experiment based on the probability theory. In this paper, the research focuses on elastic scattering of thermal neutron in the model. The thermal neutrons transporting in the medium usually may undergo one or several collisions. Between every two collisions, the neutrons would be supposed to move along a straight line without loss of energy. Therefore, it is the key step to determine a series of states during collisions in our simulation. Generally, it mainly consists of the following five steps: setting up the initial state, sampling of collision position, identifying collision atomic nucleus, determining the type of collision, and finally measuring the energy and direction of neutrons after collision.

### Calculation of the similarity and distribution of porosity

After the binary segmentation, the reconstructed model is composed of 400 × 400 × 400 pixel cubes including the matrix and the pore space. Accordingly, the size of the projected image of porosity in YZ plane is 400 × 400 pixels. For each pixel in the image, its porosity can be calculated with the 400 cubes along the direction that are perpendicular to the pixel in the three-dimensional model. Thus, the distribution of porosity in YZ plane can be obtained for a further investigation. Similarly, we can also obtain the projected distribution of porosity in XZ and XY plane respectively. In order to eliminate the impact of data magnitude, we use the same color bar to plot figures of both the porosity distribution and the counts difference, as shown in Fig. 3. Therefore, the figures can be intuitively compared with each other, and be conveniently analyzed for the similarity. After converting them into grayscale pictures represented by two-dimensional matrices, we can calculate the similarity by equation (9).


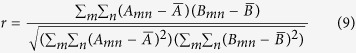


*r*: similarity;

*A*_*mn*_: the element value of matrix A;

*B*_*mn*_: the element value of matrix B;


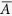
: the average value of all elements in matrix A;


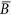
: the average value of all elements in matrix B.

### Neutron transmission imaging

As a non-destructive detection method, neutron transmission imaging method can easily detect materials composed of different elements, such as hydrogen. Besides, it is not matrix but pore-filling fluid (for example, water) that contains the largest amounts of hydrogen as constituents of the rock. Therefore, elastic reaction of thermal neutrons will mainly occur with hydrogen in the pore space. Equation (10) shows the process of monochromatic (single wavelength) neutron beam transport attenuation^3^:


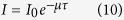


*I*: intensity of neutron beam that transported through the medium;

*I*_0_: initial intensity of incident neutron beam;

*μ*: attenuation coefficient, *cm*^−1^;

*τ*: sample thickness.

The method in each simulation is implemented according to following process: a pure matrix model of carbonate or sandstone core with zero porosity, was firstly irradiated by the thermal neutron beam. Then, put the reconstructed digital core (as shown in Fig. 1) into the same simulation environment, and conducted the simulation of thermal neutron transporting along directions of X, Y and Z, respectively. Finally, we can subtract the simulated data of the reconstructed model along different directions, from that of the pure matrix model. Thus, we can get the distribution of difference of counts between the transport of neutron in pure matrix model and that in reconstructed digital core.

## Additional Information

**How to cite this article**: Wang, Y. *et al.* Numerical research on the anisotropic transport of thermal neutron in heterogeneous porous media with micron X-ray computed tomography. *Sci. Rep.* **6**, 27488; doi: 10.1038/srep27488 (2016).

## Figures and Tables

**Figure 2:**
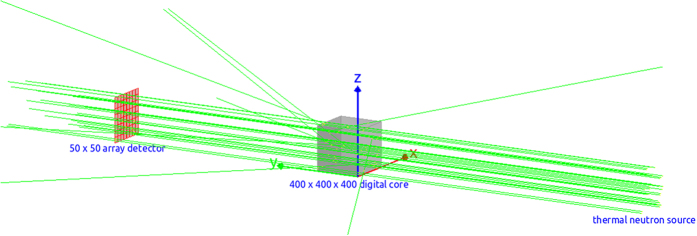
Model of thermal neutron irradiating core samples. The grey cube in the middle is the core sample; the red plane on the left is the 50 × 50 array detector; the right end of the green lines is the thermal neutron source (square surface source). The whole model is under normal atmosphere. The detector, the core model and the thermal neutron source are in the same straight line. Moreover, they have the same size in sections perpendicular to the line.

**Table 1:**
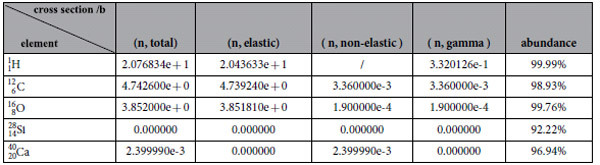
Reaction cross section of H, C, O, Si and Ca for thermal neutron and the corresponding natural abundance of isotopes^26^,^27^.

**Table 2:** Prediction of the porosity

Counts difference (*x*)	Real porosity	Relative error X	Relative error Y	Relative error Z
332780	0.053464516	0.23%	0.08%	0.38%
661348	0.108629469	−1.50%	−1.58%	−1.43%
309735	0.049879859	−0.07%	−0.23%	0.08%
426181	0.068628984	0.21%	0.09%	0.32%
